# On Certain Medico-Legal Subjects

**Published:** 1887-04

**Authors:** J. J. Abrams

**Affiliations:** Savannah (Georgia) Bar


### ON CERTAIN MEDICO-LEGAL SUBJECTS.

---

READ BEFORE THE GEORGIA MEDICAL SOCIETY AT SAVANNAH, GEORGIA, DECEMBER 7, 1886, BY J. J. ABRAMS, ESQ., OF THE SAVANNAH (GEORGIA) BAR.

---

When I undertook the pleasant task of writing an opinion upon the "Liability of physicians to attachments for contempt in refusing to testify professionally in courts," it was with the desire to endeavor, by collecting the decisions upon the subject, to enable you clearly to understand in what cases you would or could not so refuse. I had no hope of making the paper one of interest, other than that which centers around the pecuniary and legal questions involved, and should I succeed in making the dry details of law interesting to you in this sense, I shall have accomplished more than I had hoped for.

Another subject o peculiar interest to you is involved in the question of the "Civil liability of physicians and surgeons for errors committed in their practice," and as this has been suggested to me by a recent case in my own practice, I will, at the risk of this paper (being somewhat lengthy), make that subject the second portion of it, and the third subject (to which my attention has been directed by an officer of your body) is "Professional secrets, their preservation and punishment for disclosure."

First, then, "When can a physician refuse to testify professionally in a case in a court to which he has been subpœnaed?"

A physician, as such, is under no obligation to the public to testify or give his opinion on a subject with which he is peculiarly conversant, because of his profession, his skill and knowledge, derived from study and experience, are essentially his own property, and no court can deprive him of them without just compensation. In this sense, the license to practice medicine, surrounded as it is by numerous safe-guards for the public welfare, laws passed for its proper exercise, and punishments prescribed for their violation, may be termed a franchise and his exclusive property.

In the case of The People *vs.* Montgomery, 13 Abb. Pr. (N. S.), 207, Montgomery was indicted for murder, and Dr. Hammond was paid five hundred dollars as compensation for his services as an expert upon objection made. The judge (E. D. Smith), overruling the objection to the payment, said: "The district attorney, it is true, might have required the attendance of Dr. Hammond on subpœna—he could not have been required under process of subpœna—to examine the case and *to have used his skill and knowledge to enable him to give an opinion upon any point of the case*, nor to have attended during the whole trial, and attentively considered and carefully heard all the testimony given on both sides in order to qualify him to give a deliberate opinion on such testimony as an expert."

In the "Jurisprudence of medicine in its relation to the law of contracts," by John Ordronaux, sections 114 and 115, it is said: "But once upon the stand as a skilled witness his (the physician's) obligation to the public ceases, and he stands in the position of any professional man consulted in relation to a subject on which his opinion is sought."

It is evident that the skill and professional experience of a man are so far his individual capital and property that he cannot be compelled to bestow them gratuitously upon any party. Neither the public, any more than a private person, have a right to extort services from him in the line of his profession without adequate compensation. And this view of the learned writer is in accord with others.*

The courts generally seem to treat the right to practice law and the skill and knowledge employed therein as property, in a qualified sense, and the court, in Webb *vs.* Baird, 6 Ind., 13, says: "To the attorney his profession is the means of his livelihood; his legal knowledge is his capital stock; his professional services are no more at the mercy of the public, as to the remuneration, than are the goods of the merchant, or the crops of the farmer, or the wares of the mechanic.† If, then, practitioners of the law are entitled to be paid for their professional opinions as their property, a *fortiori*, so are physicians.

Ex Parte Dement (53 Ala., 389) is the only case I have been able to discover which is adverse to the above views, and in that case Dr. Dement *had seen the wounds in question*, and was asked to describe their nature, character and probable effects, declining upon the ground that he was entitled to compensation for his professional opinion. He was fined, but in that case, although the court imposed the fine, it seemed to have done so because the witness was called on to prove a fact within his knowledge, and he owed that duty to the public.

### WHEN CAN A PHYSICIAN BE COMPELLED TO TESTIFY PROFESSIONALLY?

Without reviewing the numerous adjudicated cases upon the subject, the conclusion from them may be safely stated as follows:

---

*First. Taylor's Med. Jur., page 19. Second. Phil. E. 4th., Am. Ed., page 828.

†See also Ex Parte Bennett, 12 C. L. J., 193; also Buckman *vs.* State, Supreme Court, Indiana (5 Reporter, 429.)

First, that a physician or surgeon, as such, cannot be compelled to testify to matters and things, or give an opinion upon matters derived solely from his professional skill and knowledge.

Second, that he can be punished for contempt in refusing to testify in a case where he has seen a fact and is called on to prove it in a court of justice, even though it involves professional opinions; the duty which he owes to the community in furtherance of public justice requires it of him.

A case may, I trust never will, arise in our community involving these issues, but should it so happen, we can but hope that the above conclusion will be adopted; but with the high sense of duty which pervades your learned and honorable profession, it may be that some one of you will be called on, in justice to your learned brethren, to make the issue, when the opinion given in this paper may avail you and relieve you from the imputation of refusal from contempt of authority.

### THE CIVIL LIABILITY OF PHYSICIANS AND SURGEONS FOR NEGLIGENCE.

Our Code of Laws (section 2973) upon the subject is as follows:

A person professing to practice surgery, or the administering of medicines for a compensation, must bring to the exercise of his profession a reasonable degree of care and skill.

Any injury resulting from the want of such care and skill will be a tort for which a recovery may be had.

The law, then, is certain, fixed, but what is meant by a reasonable degree of care and skill is not so certain; it may vary with the particular case calling for a decision.

For instance, in the case of a physician or surgeon who is a specialist, such as an aurist or oculist, a reasonable degree of care and skill would doubtless be construed to mean that degree of care and skill to be reasonably expected from one in his position, one holding himself out as possessing peculiar skill in these occupations.

In 4th Allen (Mass.), 268, I find this excellent definition of "reasonable care and skill:" "Reasonable care and skill is a relative phrase, and what this requires is always to be determined by consideration of the subject to which it applies, and reasonable care is synonymous with ordinary care; it is a term that has relation to the situation of the parties and business in which they are engaged."

Again, general practitioners in populous cities and towns should be held to higher degree of skill and knowledge, and hence to a higher degree of reasonable care and skill than those who live far removed from, and who do not enjoy constant opportunities for professional observation and practice. The former class have had, in addition to the theoretical knowledge, constant practice upon various diseases and accidents, while the latter are confined in their operations and practice to such cases as are furnished by the meager population by which they are surrounded, and the term, "reasonable care and skill," must, as to them, be construed in a more limited sense than is applicable to the physician in towns and cities; yet, no less than the city physician, must the care and skill of the county physician be reasonable, and it must not be measured by the care and skill exercised in the locality in which he employs it, but it must be that reasonable degree of care and skill exercised by the profession generally, without reference to the practice exercised in his locality.

Mr. Wharton, whose admirable treatise on medical jurisprudence in connection with Mr. Stille is not unknown to your profession, says: "The average skill of a professson, taking in good and bad, young and old as a mass, is difficult to reach; and if we count into the aggregate the young who have had no practice and the old who have retired from practice, the average would give a standard lower than that which should be required."

Nor is this all. Even supposing such a standard could be reached and should be adequate, it is too inflexible to be indiscriminately applied.

In a city there are many means of professional culture which are inaccessible in the country. In a city hospitals can be readily walked, and new books and appliances promptly purchased, and libraries easily visited, and in a city also exists that intercourse with prominent professional men, which leads not only to the promotion of keenness and culture, but to the free interchange of new modes of treatment. In the country such opportunities do not exist.

What is due diligence in the city is not due diligence in the country, and what is due diligence in the country is not due diligence in the city; hence the question in each particular case is to be determined, not by what would be the average diligence of the profession, but what would be the diligence of an honest, intelligent and responsible expert in the position in which the defendant was placed, and he says in section 738: "To make the physician liable it is scarcely necessary to add, his negligence must stand in immediate casual relation to the injury," the text in both sections being supported by numerous citations of respectable authority.

It is human to err, and physicians are no less human than others. If they act upon their best judgment, reasonably and fairly consistent with the standard of their profession, they are not responsible, but an error of judgment may be so gross as to display want of reasonable skill and care, and in all such cases it is believed he would be responsible.

I would not be understood as saying that the exercise of a physician's best judgment would alone be sufficient to relieve him of liability. There are and have been cases where but one mode of treatment is allowed. A radical change in such mode, although best in the judgment of the physician, is such departure as to render the physician *prima facie* liable.

In Tefft *vs.* Wilcox, 6 Kan., 46, the court say: "In stipulating to expert his skill and apply his diligence and care, the medical and other professional men contract to use their best judgment. Few cases can be supposed where but a single course of measures alone can be adopted, and many may occur where great differences of opinion may exist as to the best course to be taken.

"In most cases judgment and discretion are required to be exercised. Freedom from errors of judgment is never contracted for by the attorney or physician.

"Ordinary good judgment is necessarily implied in the possession of ordinary skill, and if such show of judgment is fairly exercised, any risk from mere error and mistakes is upon the employer alone." Doubtless many cases can be found in the books where contributory negligence on the part of the patient himself would exonerate the physician, but it is believed upon examination these decisions are based on the common law liability of practitioners and in the absence of a statute like ours.

Negligence of the patient in taking the medicine prescribed, or violating any reasonable direction or known sanitary law, cannot, of course, be want of care or skill on the part of the physician, and should not be considered in construing our Georgia statutes.

PROFESSIONAL SECRETS—THEIR PRESERVATION AND PUNISHMENT FOR DISCLOSURE.

But for the fact that this subject has been broached to me by one of your profession, I should hesitate in calling attention to it; but on reading the "Inaugural Address of Dr. R. J. Nunn, delivered before the Georgia Medical Association at Augusta, Ga.," April the 21st, 1886, I find that he mentions it as one of the matters needing attention from the profession; therefore I may be pardoned for expressing an opinion upon a subject of professional interest to you.

Upon referring to authorities on this subject, I find that communications of this character are not regarded in law as privileged in the absence of statutory enactments, and while the tendency is to assert the inviolability of such secrets, the matter has been much discussed, whether, under the Roman common law, a physician is privileged as to matters confidentially imparted to him by a patient. But certain it is that in modern times the sacredness of professional secrets is held in higher regard, as is evidenced by the passage of statutes in several States prohibiting under a penalty their disclosure.

It were needless for me to expatiate upon the confidential character of communications between physician and patient. Next to those of the penitent and confessor, there is no relation which should be regarded as more sacred. What number of households might be wrecked; what misery entailed upon the living and the unborn, and reflections upon the memory of the dead, through even unwitting disclosures of such secrets, it is needless for me to dwell on; and if such unwitting utterance should work these results, how much worse would the consequences be if a physician is allowed by law to make such disclosures? He should not be permitted to do so, and an entire prohibition—so far as punishment can prohibit—should be put upon such disclosures.

By the statutes of Georgia (Code of 1882, section 3797, sub-div. 2), "There are certain admissions and communications excluded from public policy; among these are those "between counsel and client." Why should these communications, involving as they do in most cases mere money demands, be excluded from public policy?

It is because of the confidence reposed in the attorney and the nature of the communications; and in Woods *vs.* Woods, 4 Hare, 83, Sir James Wigram thus briefly states the necessity for the rule: "So long as the state of the law shall make it impossible for parties to be their own lawyers, and act without professional advice, it is indispensably necessary that the privileges now conceded to professional communications should be maintained." And Lord Brougham, in Greenough *vs.* Gaskell I. Myle and Keen's Chancery Report, p. 98, says:

"To force from the party himself the production of communications made by him to the professional men seems inconsistent with the possibility of an ignorant man resorting to professional advice, and can only be justified if the authority of decided cases warrants it. But no authority sanctions the much wilder violation of professional confidence, and in circumstances wholly different which would be involved in compelling counsel, or attorneys, or solicitors, to disclose matters committed to them in their professional capacity, and which but for their employment as professional men they would not have become possessed of," why should not this reasoning be applied to your profession?

If such great judicial minds as Judge Brougham recognized the importance of preserving inviolate the sacredness of commucations between attorney and client, can you not be pardoned for wishing to likewise place the seal of secrecy upon communications between physician and patient—secrets the nature of which are oftentimes of a more sacred character, involving the good name and fair fame of its communicant, the happiness and future of the innocent and the preservation of family ties, the dissolution of which by disclosure of them might entail suffering upon many? It is no reflection upon your integrity as professional men to ask legislation upon the subject. It is to extend the protection of the law to your patients, and for their protection, that legislation in this State is needed; and as to the penal enactment, its preventive force can be applied to those of your professional brethren who have not that high sense of honor and regard for professional ethics, and "whose tongues are their own shame's orator." For it cannot be successfully denied that into all professions there sometimes creep men whose discretion, no less than their professional skill, cannot be relied on, and to this class of persons the law will apply and protect the patient from.

I would advise you, therefore, to ask the present legislature to place among the class of privileged communications those between physician and patient, in the language of the statutes of New York, which is as follows: "No person duly authorized to practice physic or surgery shall be allowed to disclose any information which he may have acquired in attending any patient in a professional character, and which information was necessary to enable him to prescribe for the patient as a physician, or do any act for him as a surgeon, whether such information be disclosed by the patient or found by the physician or surgeon on examination," and this might also be added to the penal laws and punished as a misdemeanor, the penalty being fine or imprisonment, or both, at the discretion of the court.

